# The Emerging Role of MicroRNA-155 in Cardiovascular Diseases

**DOI:** 10.1155/2016/9869208

**Published:** 2016-11-27

**Authors:** Richard Y. Cao, Qing Li, Yi Miao, Ying Zhang, Wenchao Yuan, Li Fan, Gongliang Liu, Qiongyao Mi, Jian Yang

**Affiliations:** ^1^Laboratory of Immunology, Shanghai Xuhui Central Hospital/Shanghai Zhongshan-Xuhui Hospital, Fudan University/Shanghai Clinical Research Center, Chinese Academy of Sciences, 966 Middle Huaihai Road, Shanghai 200031, China; ^2^Department of Rehabilitation, Shanghai Xuhui Central Hospital/Shanghai Zhongshan-Xuhui Hospital, Fudan University/Shanghai Clinical Research Center, Chinese Academy of Sciences, 966 Middle Huaihai Road, Shanghai 200031, China; ^3^Clinical Laboratory, Shanghai Xuhui Central Hospital/Shanghai Zhongshan-Xuhui Hospital, Fudan University/Shanghai Clinical Research Center, Chinese Academy of Sciences, 966 Middle Huaihai Road, Shanghai 200031, China; ^4^School of Life Sciences, Shanghai University, 99 Shangda Road, Shanghai 200444, China

## Abstract

MicroRNAs have been demonstrated to be involved in human diseases, including cardiovascular diseases. Growing evidences suggest that microRNA-155, a typical multifunctional microRNA, plays a crucial role in hematopoietic lineage differentiation, immunity, inflammation, viral infections, and vascular remodeling, which is linked to cardiovascular diseases such as coronary artery disease, abdominal aortic aneurysm, heart failure, and diabetic heart disease. The effects of microRNA-155 in different cell types through different target genes result in different mechanisms in diseases. MicroRNA-155 has been intensively studied in atherosclerosis and coronary artery disease. Contradictory results of microRNA-155 either promoting or preventing the pathophysiological process of atherosclerosis illustrate the complexity of this pleiotropic molecule. Therefore, more comprehensive studies of the underlying mechanisms of microRNA-155 involvement in cardiovascular diseases are required. Furthermore, a recent clinical trial of Miravirsen targeting microRNA-122 sheds light on exploiting microRNA-155 as a novel target to develop effective therapeutic strategies for cardiovascular diseases in the near future.

## 1. Introduction

Cardiovascular diseases involve the heart and/or blood vessels. Despite dramatic diagnostic and therapeutic advances, cardiovascular diseases still remain the leading cause of death globally [1]. Understanding the underlying molecular and cellular mechanisms may contribute to the prevention of cardiovascular diseases.

MicroRNA (miRNA), about 22 nucleotides in length, was first discovered to regulate the* C. elegans* heterochronic gene lin-14 and further found to function in RNA silencing and posttranscriptional regulation of gene expression by binding to specific sites in the 3′ untranslated region of their target mRNAs [2–4]. A single miRNA is able to downregulate the expression of numerous target genes, so that a single miRNA can regulate complex pathophysiological processes. MiRNAs have been shown to be involved in cardiovascular remodeling [5, 6], which results in cardiovascular diseases such as coronary artery disease (CAD), abdominal aortic aneurysm (AAA), and heart failure (HF). Many miRNAs play a role in some aspects of cardiovascular remodeling, for example, miRNA-21-3p in sepsis-associated cardiac dysfunction, miRNA-433 in cardiac fibrosis, miRNA-33 and miRNA-145/143 in atherosclerosis, miRNA-21 and miRNA-320 in CAD, and miRNA-1 and miRNA-133 in HF [7–10], while miRNA-222 protects against pathological cardiac remodeling [11].

MiRNA-155 is transcribed from the B-cell integration cluster (BIC) that is located on chromosome 21 [12]. Although miRNA-155 was first discovered in children with Burkitt Lymphoma [13] and further found to act as an oncogene or a tumor suppressor in different types of cancer [14], growing evidences suggest that miRNA-155 has been considered as an important pleiotropic regulator of cell homeostasis and a typical multifunctional miRNA that regulates multiple pathophysiological pathways in hematopoietic lineage differentiation, immunity, inflammation, viral infections, and cardiovascular diseases [15, 16]. Luciferase report assays demonstrated that miRNA-155 could bind directly to the 3′ UTR of angiotensin II receptor type 1 (AGTR1), which was associated with aneurysm formation [17, 18]. To fully elucidate the involvement of miRNA-155 in cardiovascular diseases, a review is given here to discuss the emerging role of miRNA-155 in CAD, aneurysm formation, HF, and diabetic heart disease (DHD).

## 2. MiRNA-155 in CAD

CAD, secondary to coronary atherosclerosis, also known as ischemic heart disease, is the most common type of cardiovascular disease and leading cause of death globally. It includes stable angina, unstable angina, myocardial infarction, and sudden cardiac death [19]. The association between CAD and miRNA-155 has been increasingly studied, but the results are not consistent.

A high-throughput array screening of 667 miRNAs was conducted in patients who survived acute myocardial infarction (MI). MiRNA-155 was among the 11 miRNAs highly expressed in sera of patients at high risk for cardiac death. Thus, miRNA-155 might be a prognostic marker for cardiac death in post-MI patients [20]. Decreased serum levels of miRNA-155, along with increased target gene SH2-containing inositol 5′-phosphatase 1 (SHIP-1) expression was associated with reduced incidence of periprocedural MI, lower level of cardiac troponin I, and less inflammatory cytokine (INF-*γ*, TNF-*α*, and IL-6) expression after rosuvastatin treatment in post-percutaneous coronary intervention (PCI) patients with acute coronary syndromes (ACS). Therefore, the beneficial effect of rosuvastatin might be explained in part by suppression of the miRNA-155/SHIP-1 signaling pathway [21]. Tian et al. observed that the increase of miRNA-155 in CD14^+^ monocytes from patients with CAD was associated with the elevation of proatherosclerotic factors TNF-*α* and IL-6, which might affect monocytes [22]. These clinical findings were further confirmed by basic research in animal models. Tian et al. further demonstrated that miRNA-155 promoted foam cell formation through targeting of HMG box-transcription protein 1 (HBP1) in atherosclerosis *ApoE* ^−/−^ mice. Eisenhardt and colleagues found that upregulated miRNA-155 accompanied with downregulated target gene suppressor of cytokine signaling 1 (SOCS-1) was correlated with increased expression of TNF-*α*, IL-1*β*, CD105, Caspase 3, and leukocyte infiltration [23]. A German research team demonstrated that miRNA-155 was specifically expressed in atherosclerotic plaques and proinflammatory macrophages and promoted atherosclerosis by the mechanism of repressing target gene B-cell lymphoma 6 protein (BCL6) expression in atherosclerotic mice [24].

Nevertheless, opposite results were observed in clinical studies. Yao et al. found that miRNA-155 was decreased in patients with ACS, which was inversely correlated with Th17 cell differentiation [25]. In a study of 50 patients with MI, miRNA-155 from infarcted heart tissue was found to be downregulated in the ventricular rupture group, a more severe condition, in comparison with patients without ventricular rupture [26]. In a small sample study of three patients with early coronary atherosclerosis, miRNA-155 was also found to be downregulated in plaques [27]. Another study in 12 patients who died from ACS got similar results: miRNA-155 was downregulated in coronary arteries [28]. Zhu et al. reported that miRNA-155 expression measured by RT-PCR was significantly lower in 56 patients with CAD than those in 54 controls [29].

Moreover, some researchers reported that elevated miRNA-155 did not contribute to CAD. Li and colleagues found that miRNA-155 in foam cells and clinical specimens from patients with atherosclerosis was significantly elevated, but they thought that increased miRNA-155 targeting calcium-regulated heat stable protein 1 (CARHSP1) played a protective role during atherosclerosis-associated foam cell formation [30]. Zhu's research group also showed significant elevation of miRNA-155 in both thoracic aorta from ApoE^−/−^ mice and plasma from patients with CAD. They believed that increased miRNA-155 contributed to the prevention of atherosclerosis development via targeting mitogen-activated protein kinase kinase kinase 10 (MAP3K10) [31].

Above all, miRNA-155 was found to be either upregulated or downregulated in CAD patients and thus might promote or prevent CAD. These conflicting results of miRNA-155 in the pathophysiology of atherosclerosis related ischemic heart diseases indicate the complexity of this multifunctional molecule in regulation of cardiovascular remodeling induced by atherogenesis. The presumable causes of this inconsistence might be due to different pathological stages of the disease. Therefore, more comprehensive studies of underlying mechanisms of miRNA-155 involvement in CAD are needed.

## 3. MiRNA-155 in AAA

AAA is defined by a localized dilation of the abdominal aorta exceeding the normal diameter by more than 50%. AAA is a common cardiovascular disease with life-threatening implication from aortic rupture [32, 33]. The risk factors of AAA are associated with advanced age, male gender, cigarette smoking, atherosclerosis, hypertension, and genetic predispositions [34].

Using the FlexmiR™ MicroRNA Assay to screen 124 miRNAs within AAA biopsies and sera of 10 patients undergoing AAA repair in comparison with 10 age- and sex-matched controls, and further confirming the screening results by qRT-PCR, 7 miRNAs were found to be upregulated [35]. In AAA biopsies, miRNA-155 was the most highly expressed: about 11.32-fold higher than control. Serum miRNA-155 had a 2.67-fold increase in AAA patients compared with controls. Two miRNA-155 target genes cytotoxic T-lymphocyte-associated protein 4 (CTLA4) and mothers against decapentaplegic homolog 2 (SMAD2) were both found to be significantly downregulated within AAA biopsies. These findings suggested that miRNA-155 was overexpressed in aneurysmal tissues with implications in the pathogenesis of AAA. However, another screening of miRNAs in both AAA tissues and plasma from patients undergoing AAA repair showed that miRNA-155 was upregulated in AAA tissues but downregulated in plasma in comparison with controls [36]. In situ miRNA-155 in aneurysmal tissue does not match systemic changes in peripheral blood, implying unparalleled regulation of miRNA-155 in aortic aneurysm disease. Therefore, understanding the molecular mechanisms of miRNA-155 in AAA pathogenesis becomes extraordinarily important.

## 4. MiRNA-155 in HF

HF, usually referred to as congestive heart failure, is a complex clinical syndrome of signs and symptoms that suggest impairment of the heart to pump sufficiently to meet the body needs. The most common symptoms include shortness of breath, fatigue, and ankle swelling. HF is caused by structural or functional abnormalities of the heart such as CAD, hypertension, atrial fibrillation, infection, and cardiomyopathy [37]. HF is the leading cause of hospitalization with immense health and economic burdens [38]. To determine the involvement of miRNAs in HF, 1105 miRNAs were screened by GeneChio miRNA 2.0 Array from blood samples in a study of 42 HF patients and 15 age- and sex-matched healthy volunteers [39]. Among the 1105 screened miRNAs, 29 showed significant dysregulation, and 8 of the 29 were upregulated in sera of HF patients compared to healthy controls. Notably, miRNA-155 was not only upregulated but also positively correlated with left ventricular mass index, a prognostic marker in HF patients. Consistent with the above results, Marques et al. showed that miRNA-155 was upregulated in HF patients after screening 84 cardio-miRNAs in blood samples from 9 patients with HF and 8 healthy volunteers [40].

A laboratory study confirmed the role of miRNA-155 in pathological cardiac remodeling, one of the important causes of HF, in which loss of miRNA-155 in fibroblasts protected left ventricular function after experimental acute myocardial infarction [41]. To elucidate the molecular mechanisms, this study further demonstrated that miRNA-155 knockout improved cardiac remodeling through targeting tumor protein p53-inducible nuclear protein 1 (TP53INP1). Thus, clinical screenings and findings of miRNA-155 involvement in HF patients were proved by basic research in transgenic animals.

## 5. MiRNA-155 in DHD

Diabetes mellitus (or simply called diabetes) is a metabolic disorder with chronic hyperglycemia resulting from defects in insulin secretion and/or action. Symptoms of diabetes include polydipsia, polyuria, and polyphagia [42]. If not treated properly, diabetes can cause serious complications such as cardiovascular disease, stroke, chronic kidney failure, foot ulcers, and eye problems. Diabetes increases the risk for heart disease. DHD refers to heart disease that develops in people who have diabetes. DHD may include CAD, HF, and diabetic cardiomyopathy [43]. Diabetes is a major and independent predictor of subsequent heart failure in patients after MI [44]. In a large investigation of 1088 miRNAs in left ventricular specimens from streptozotocin-induced diabetic mice, 316 miRNAs were found to be dysregulated. Of those 316 miRNAs, miRNA-155 was among 268 miRNAs that remained significantly altered in heart tissues of diabetic mice even though normoglycaemia was reached subsequently [45]. In a mouse model of diabetic MI, Kishore and colleagues demonstrated that bone marrow-derived progenitor cell therapy contributed to prevention of cardiac fibrosis after MI by releasing hepatocyte growth factor, which inhibited miRNA-155-*Ski* (Sloan-Kettering Institute proto-oncogene)/*SnoN* (Ski-related novel gene, non-Alu-containing) mediated profibrosis signaling pathway. Their data suggested that inhibition of miRNA-155 might protect against cardiac fibrosis in the diabetic heart [46].

## 6. Conclusion

In summary, this review integrates published materials of current research on miRNA-155 that have long been associated with cardiovascular diseases. Both clinical and laboratory research data indicate an emerging role of miRNA-155 and its target genes (Table 1) in cardiovascular diseases, including CAD, AAA, HF, and DHD. The effects of miRNA-155 in different cell types through different target genes result in different mechanisms in diseases. For example, miRNA-155 was specifically expressed in proinflammatory macrophages and promoted foam cell formation through miRNA-155-*HBP1* signaling pathway in atherosclerosis [22]. Endothelial* AGTR1* was the earliest confirmed miRNA-155 target gene [47], which contributed to AAA formation [18]. MiRNA-155 knockout in fibroblasts improved cardiac remodeling, one of the important causes of HF, through targeting* TP53INP1* gene [41], while inhibition of miRNA-155 in fibroblast through its target genes* Ski* and* SnoN* might protect against cardiac fibrosis in the diabetic heart [46].

MiRNA-155 is intensively studied in relation to atherosclerosis and CAD. However, miRNA-155, as a multifunctional molecule, is conflictingly reported to be either upregulated or downregulated in atherosclerosis of CAD patients. MiRNA-155 has also been demonstrated to play contradictory roles by either promoting or preventing the pathophysiological process of atherogenesis, associated with cardiovascular remodeling and ischemic heart diseases. These inconsistent results warrant further investigations.

Experimental inhibition of miRNA-155 attenuated myocardial damage during acute myocarditis and thus might be a potential therapeutic target for viral myocarditis [48]. The clinical study of rosuvastatin treatment in 159 patients with ACS after PCI demonstrated that rosuvastatin reduced the incidence of cardiovascular events through suppressing miRNA-155-*SHIP-1* molecular signaling pathway [21]. Moreover, the first clinical trial targeting miRNA-122 using Miravirsen, an antisense oligonucleotide against hepatitis C virus [49], sheds light on clinical application of miRNA inhibitors. Taken together, the aforementioned examples offer the possibility of potentially exploiting miRNA-155 as novel targets to develop effective therapeutic strategies for cardiovascular diseases in the near future. Clinical trials and further extensive studies from bench to bedside are required to reveal the clinical and mechanistic roles of miRNA-155 in the course of atherosclerotic development correlates with different types and stages of CAD as well as other cardiovascular diseases such as AAA, HF, and DHD.

## Figures and Tables

**Table 1:** MiRNA-155 and target genes in cardiovascular diseases.

Diseases	Target genes	References
CAD	*SHIP1*, *HBP1*, *SOCS1*, *BCL6*, *CARHSP1*, *MAP3K10*	[21–24, 30, 31]
AAA	*AGTR1*, *CTLA4*, *SMAD2*	[17, 35]
HF	*TP53INP1*	[41]
DBH	*Ski*, *SnoN*	[46]

AAA: abdominal aortic aneurysm; AGTR1: angiotensin II receptor 1; CAD: coronary arterial disease; DBH: diabetic heart; HF: heart failure; BCL6: B-cell lymphoma 6; CARHSP1: calcium-regulated heat stable protein; CTLA4: cytotoxic T-lymphocyte-associated protein 4; HBP1: HMG-box transcription factor 1; HGF: hepatocyte growth factor; MAP3K10: mitogen-activated protein kinase kinase kinase 10; SHIP1: SH2-containing inositol 5′-phosphatase 1; Ski: Sloan-Kettering Institute proto-oncogene; SMAD2: mothers against decapentaplegic homolog 2; SnoN: Ski-related novel gene, non-Alu-containing; SOCS1: suppressor of cytokine signaling 1; TP53INP1: tumor protein p53-inducible nuclear protein 1.
